# Cost-Effectiveness of a New Outpatient Pulmonology Care Model Based on Physician-to-Physician Electronic Consultation

**DOI:** 10.1155/2022/2423272

**Published:** 2022-10-31

**Authors:** José Manuel Álvarez-Dobaño, Malena Toubes, José Ángel Novo-Platas, Francisco Reyes-Santías, Gerardo Atienza, Manuel Portela, Carlos Rábade, Tamara Lourido, Ana Casal, Carlota Rodríguez-García, Vanessa Riveiro, Romina Abelleira, Jorge Ricoy, Nuria Rodríguez-Núñez, Carlos Zamarrón, Felipe Calle, Francisco Gude, Luis Valdés

**Affiliations:** ^1^Servicio de Neumología, Complejo Hospitalario Clínico-Universitario de Santiago, Grupo Interdisciplinar de Investigación en Neumología, Instituto de Investigación Sanitaria de Santiago de Compostela (IDIS), Santiago de Compostela, Spain; ^2^Servicio de Neumología, Complejo Hospitalario Clínico-Universitario de Santiago, Santiago de Compostela, Spain; ^3^Servicio de Control de Gestión, Área Sanitaria de Santiago de Compostela y Barbanza, Complejo Hospitalario Clínico-Universitario de Santiago, Santiago de Compostela, Spain; ^4^Complejo Hospitalario Universitario de Santiago, Santiago de Compostela, Spain; ^5^Unidad de Calidad y Seguridad del Paciente, Subdirección de Calidad, Gerencia Área Sanitaria de Santiago de Compostela y Barbanza, Complejo Hospitalario Universitario de Santiago, Santiago de Compostela, Spain; ^6^Subdirección de Prestación Farmacéutica Formación Docencia Innovación e Investigación, Gerencia Área Sanitaria de Santiago de Compostela y Barbanza, Instituto de Investigación Sanitaria de Santiago de Compostela (IDIS), Santiago de Compostela, Spain; ^7^Subdirección de Processos Asistenciales Sin Ingresso, Gerencia Área Sanitaria de Santiago de Compostela y Barbanza, Complejo Hospitalario Universitario de Santiago, Santiago de Compostela, Spain; ^8^Unidad de Epidemiología Clínica, Complejo Hospitalario Clínico-Universitario de Santiago, Santiago de Compostela, Spain

## Abstract

**Introduction:**

This study assesses the impact of an electronic physician-to-physician consultation program on the waiting list and the costs of a Pulmonology Unit.

**Materials and Methods:**

A prepost intervention study was conducted after a new ambulatory pulmonary care protocol was implemented and the capacity of the unit was adopted. In the new model, physicians at all levels of healthcare send electronic consultations to specialists.

**Results:**

In the preintervention year (2019), the Unit of Pulmonology attended 7,055 consultations (466 e-consultations and 6,589 first face-to-face visits), which decreased to 6,157 (3,934 e-consultations and 2,223 first face-to-face visits; 12.7% reduction) in the postintervention year (all were e-consultations). The mean wait time for the first appointment was 25.7 days in 2019 versus 3.2 days in 2021 (*p* < 0.001). In total, 43.5% of cases were solved via physician-to-physiciane-consultation. A total of 2,223 patients needed a face-to-face visit, with a mean wait time of 7.5 days. The mean of patients in the waiting listing decreased from 450.8 in 2019 to 44.8 in 2021 (90% reduction). The annual time devoted to e-consultations and first face-to-face visits following an e-consultation diminished significantly after the intervention (1,724 hours versus 2,312.8; 25.4% reduction). Each query solved via e-consultation represented a saving of €652.8, resulting in a total annual saving of €827,062.

**Conclusions:**

Physician-to-physiciane-consultations reduce waiting times, improve access of complex patients to specialty care, and ensure that cases are managed at the appropriate level. E-consultation reduces costs, which benefits both, society and the healthcare system.

## 1. Introduction

A public health system must be built on the principles of equity by providing services to all patients irrespective of their economic or social status; effectiveness, by solving health problems at the adequate level of healthcare and preventing unnecessary medical procedures and unneeded complementary tests studies; quality, by using quality indicators to assess clinical activity and health outcomes and identify dysfunctions; and transparency, by making results public [1, 2]. To attain these goals, it is necessary to exploit information systems and promote the implementation of collaborative networks at the different levels of care [3], which will ultimately guarantee the sustainability of the system [4].

Waiting lists are one of the most important challenges that prevent our public health system from complying with these principles. Before the intervention, the volume of outpatient consultations had increased in our third-level hospital Unit of Pulmonology. This increase was partially due to the interventions implemented to reduce the number of hospital admissions (one-stop consultations, day hospital, and outpatient management of patients undergoing invasive diagnostic procedures, to name a few). Although waiting lists are unavoidable, long waitlists may compromise the principle of equity and raise ethical concerns about distributive justice, inequalities, or discrimination [5]. Telemedicine based on electronic physician-to-physician consultations (e-consultations) may help reduce waiting lists, thereby improving healthcare equity, effectiveness, and clinical outcomes [6]. Through e-consultations, physicians provide clinical information and send consultations securely to specialists (in this case, a pulmonologist), via the electronic health record of patients with good reported results in other specialties [6, 7]. By this method, the specialist decides whether a patient needs a face-to-face appointment with specialty care, or otherwise the case can be managed in primary healthcare (PH) following the specialist's advice.

The purpose of this study was to assess the effects of a physician-to-physiciane-consultation model on wait times and costs associated with patient referrals from primary or specialty care to the Pulmonology Unit of a third-level hospital in Spain.

## 2. Materials and Methods

The study was conducted in a tertiary 1000-bed university hospital serving a population of 450,000 distributed across 46 city councils, with 24.2% of the population being older than 65 years. For the population older than 14, there are 301 general practitioners (GPs) distributed across 56 primary healthcare centers and 21 rural clinics.

In this pre and postintervention study (implementation of a physician-to-physiciane-consultation model), the year 2019 corresponds to the preintervention period, whereas 2021 corresponds to the postintervention period. The year 2020 was considered as the “washout” period, since e-consultations started in June 2020, the two models overlapped during the first weeks, and the outbreak of the COVID-19 pandemic would have influenced the results obtained.

In 2019, the Pulmonology Unit had 15 open slots per weeks for e-consultations and 156 slots per week for first face-to-face appointments. In 2020, an analysis was performed of the results of 2019 to estimate the resources that were necessary to implement the e-consultation model. The capacity of the Pulmonology Unit increased to 157 e-consultations and 96 first face-to-face consultations per week (44 weeks/year). The estimated mean time devoted to an e-consultation and first face-to-face visit was 15′ and 20′, respectively (both, for the pre and the postintervention period).

Until 2019, GPs were given the option to choose between a conventional face-to-face visit or an e-consultation. From June 1^st^ 2020, GPs and specialty physicians could only refer patients to the Unit of Pulmonology via e-consultation. The established protocol is as follows: an initial telematic evaluation (e-consultation) is performed to sort out patients with mild conditions from patients with severe diseases. The pulmonologist has to respond to the e-consultation within 3 days. The pulmonologist refers patients with mild conditions back to their referring physician via another e-consultation indicating a treatment plan (management of stable patients or with minor symptoms).

**Figure 1:**
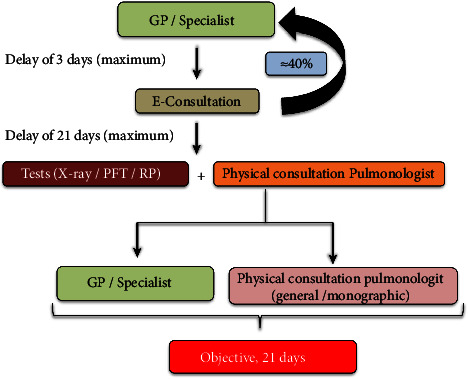
Referral protocol to outpatient pulmonology care since June 2020. Legend. PFT: pulmonary function test; GP: general practitioner; RP: respiratory polygraphy; and X-ray: chest X-ray.

It was estimated that 40% of cases would be solved via e-consultation. If a patient needed a face-to-face visit, the pulmonologist could also order, where appropriate, a chest X-ray, blood test, a repeat lung function test to complement the studies performed in PH (FeNO, CO diffusion, and static lung volumes), and/or respiratory polygraphy. For this model to be effective, coordination was required with the Department of Pulmonary Function Testing, the Sleep Unit, and the Department of Radiology. Thus, since these departments were potential bottlenecks of the new model [8], some slots were reserved for basic studies. The face-to-face visit, along with the complementary studies requested, has to be scheduled for a suitable date based on whether the patient is preferential or not. Face-to-face appointments, including appointments for complementary tests, had to be scheduled within 3 weeks after the e-consultation. Then, the pulmonologist can either refer the patient back to the referring physician or manage the case directly in the Pulmonology Unit. Finally, outpatient care agendas were adapted to the estimated demand based on data from previous years.  Figure 1 shows the e-consultation protocol. Waiting time (in days) was established according to the date of consultation considered appropriate by the referring physician. In case such a date was not detailed on the electronic request, it was calculated from the day after the date of request.

Costs were estimated based on (i) the official fees published by the Servicio Gallego de Salud [9] (40% lower for e-consultations), multiplied by 0.19 €/Km [10] if the patient was referred to the hospital [11]; (ii) time to travel to the hospital including the duration of the visit (weighted average time to reach the hospital, park the car, reach the pulmonology ward, and time spent in the face-to-face visit (79′)); (iii) costs depending on whether the population was active [based on patient average gross income (€18,768.21) and unemployment rate (7.9%)] [12]; or retired (2.3% of subjects >65 are engaged in volunteer work [13] and 22.6% perform activities that contribute to the gross domestic product) [14]; and (iv) costs related to leisure time (47% of labor cost) [15], wait time (101.5 €/month) [16], and CO_2_ emissions per private car travel (volume of CO_2_ emitted per kilometer and cost of each CO_2_ gram) [17–20].

## 3. Statistical Analysis

Continuous variables are expressed as mean ± standard deviation or as median values [interquartile interval] for asymmetric distributions. *X*^2^ and Mann–Whitney *U* tests were used to analyze the differences between consultation models. Data analysis were performed using SPSS version 22.0 (SPSS Inc., USA).

## 4. Results

**Figure 2:**
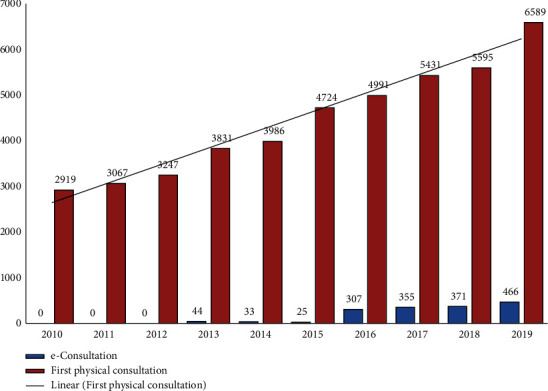
Evolution of requests for e-consultations and first face-to-face consultations to pulmonology (years 2010–2019). Legend of figure: columns in maroon correspond to face-to-face consultations.

Figure 2 shows the evolution of the number of first-visit requests and e-consultations in the Unit of Pulmonology between 2010 and 2019. The total number of first appointments increased by 125.7% during this period.

**Figure 3:**
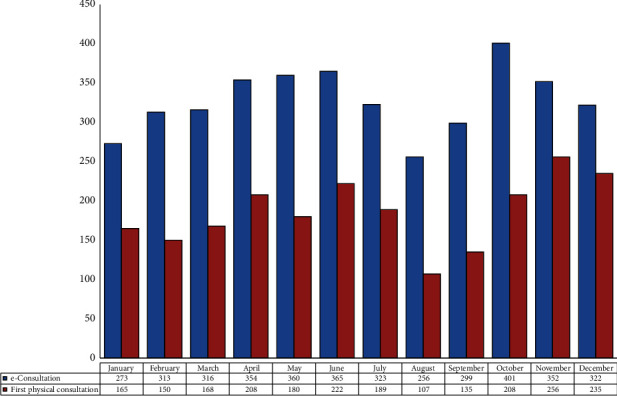
Monthly number of e-consultations and first face-to-face consultations in 2021.

A total of 7,055 consultations (466 e-consultations and 6,589 first face-to-face visits) were performed in the preintervention year (466 e-consultations and 6,589 first face-to-face visits) (Figure 2). In the postintervention year, 3,934 e-consultations were conducted (44.2% reduction), which generated 2,223 first face-to-face visits (12.7% reduction).  Figure 3 details the monthly distribution of e-consultations and the associated first face-to-face visits conducted in 2021. On average, there were 327.8 e-consultations (range, 256–401) and 185.3 face-to-face visits (range, 107–256) per month. As many as 43.5% of cases were solved via e-consultation.

Table 1 describes the clinical-epidemiological characteristics of patients seen in 2019 and 2021. There were no statistically significant differences in terms of age, sex, or any of the associated comorbidities.

**Figure 4:**
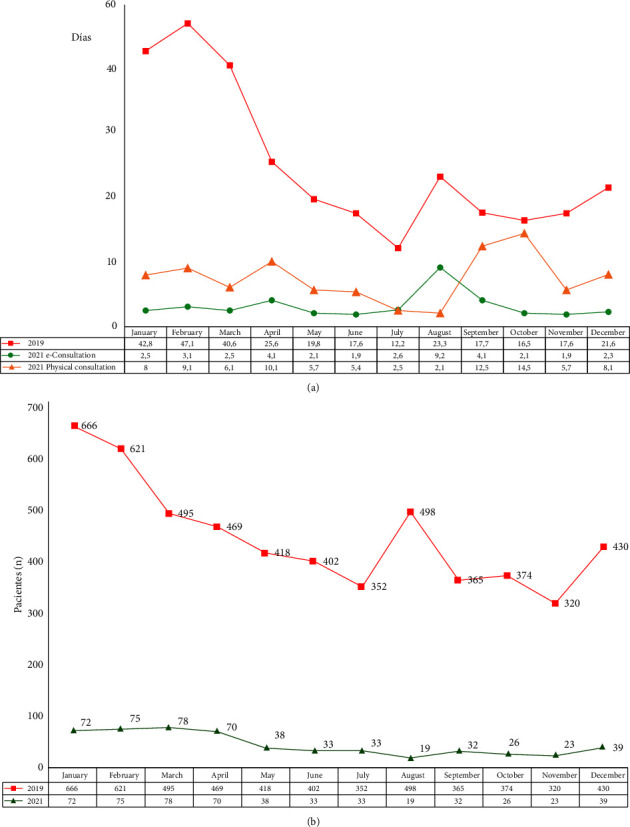
(a) Evolution of monthly mean wait time for first appointment (2019), e-consultations, and first face-to-face consultation. (b) Evolution of the number of patients in the waiting list for the first appointment with the pulmonologist by months (2019 and 2021).

Figure 4(a) shows the evolution of the mean monthly waiting time for the first appointment (2019), e-consultation, and first face-to-face visit (2021). In 2019, the mean wait time was 25.7 days vs. 3.2 (*p* < 0.001) for e-consultations. The mean wait time for the first face-to-face visit exceeded the suitable date by 7.5 days.  Figure 4(b) shows the number of patients on the waiting list for a first visit to the Unit of Pulmonology on the last day of every month. In 2019, there were 450.8 (range, 320–666) patients on the waiting list in 2019 vs. 44.8 in 2021 (range, 19–72) (90% reduction).

Table 2 shows the annual time spent by our pulmonologists on e-consultations and face-to-face visits in 2019 and 2021. In the preintervention year, the total time devoted to consultations was 2,312.8 hours. In the postintervention year, although the capacity of the unit had been increased, the total time spent on this type of consultation was 1,724.5 hours (25.4% reduction).

Table 3 summarizes the results of the economic study, which reveals that each query that is solved with e-consultation results in a saving of €652.8. Considering that 56.5% of patients required a face-to-face appointment and the mean wait time was 7.5 days vs. 25.7 days for conventional consultations, the e-consultation program represented a saving of €827,061.6.

## 5. Discussion

The results obtained suggest that our outpatient pulmonology care model based on an initial evaluation through an e-consultation reduces waiting time significantly. This model makes it possible to sort out less complex cases that do not require a face-to-face visit; this way, waiting times are reduced for more complex cases that actually need specialty care. As compared to face-to-face visits, e-consultations result in a cost reduction, which benefits both, the healthcare system and society.

In early 2020, the situation in the Outpatient Unit of Pulmonology was concerning, as the demand for first appointments had increased by 125.7% in the last decade, and the mean waiting time for the first appointment was 25.7 days in the previous year (monthly range: 12.2–47.1). This situation was unacceptable for patients and could cause the collapse of outpatient pulmonology services. Although waiting time can be reduced by reinforcing staff, this would not have diminished the sustained demand increase. By only hiring more staff, we would have faced the same problem in a few years. In conventional outpatient care, a case is not actually evaluated until the first face-to-face visit. If during the first visit the physician requests complementary studies to establish a diagnosis and/or assess disease severity, the results will not be available until the following visit some weeks later. This model delays the final diagnosis and initiation of treatment. This renders the model clearly inefficient.

According to Oseran and Wasf [21], e-consultations should facilitate physician-to-physician communication that allow asynchronous communication, enable requests and responses to be made via a secure electronic system and recorded on the patient's medical record, and address a specific health problem. Our e-consultation model meets all criteria.

The implementation of this model reduced significantly the mean waiting time. Although there are previous experiences in other medical specialties, to the best of our knowledge, this is the first time an e-consultation model is applied to pulmonology [22–27]. In total, 43.5% of cases were solved via e-consultation, slightly exceeding our estimates, and in an intermediate range with respect to previous studies (range: 21.4–69%) [6, 22, 23]. The proportion of cases solved via e-consultation is expected to decrease over the years as a result of two effects: learning, as clinicians will progressively improve their patient management skills; and dissuasive, due to an increasing awareness that minor respiratory problems are rarely referred to a face-to-face consult. This would partially explain the reduction of referrals observed after the implementation of the e-consultation model. If our estimates are correct, the number of annual e-consultations will decrease, as it already occurred during the first year of intervention. In addition, the number of face-to-face appointments following an e-consultation should remain stable, since referral criteria will not presumably change over time. This way, demand for outpatient care will progressively stabilize, which will make it easy to estimate the annual demand for face-to-face appointments and calculate the number of resources required for this type of consultation.

Our results suggest that e-consultations prevent unnecessary actions, foster the management of health problems at the appropriate level of healthcare, and possibly reduce costs by avoiding unnecessary medical procedures, complementary studies, and travel [21, 24, 25]. This contributes to ensuring the sustainability of the healthcare system. In addition, this model facilitates the provision of timely specialty care to patients with severe conditions and optimizes the use of resources. The reduction in the mean wait time achieved in our study is consistent with that reported in previous studies, although they were performed in a Unit of Cardiology [26]. In a similar study, Winchester et al. [26] achieved to reduce the wait time for the first appointment from 24 to 13 days (46%) and estimated that 60% of cases could be managed via e-consultation since they were related to patients with stable disease or mild symptoms. Comparison of results is not possible since, to the best of our knowledge, there are no previous experiences in other units of pulmonology in the literature. Of the 21 studies analyzed in a systematic review, 6 involved patients with heart problems, whereas the remaining 15 involved patients of different specialties [21].

The reduction in waiting time could be partially attributed to the increased capacity of the Unit of Pulmonology. This is theoretically true, although the total time spent on consultations was significantly lower in the postintervention year, as compared to the preintervention year (1,724.5 hours vs. 2,312.8, respectively; 25.4% reduction). This can be explained by the fact that the total number of consultations was lower in the postintervention year and dramatically below the number of consultations expected for 2021 (3,109 hours). Nevertheless, if the number of consultations had grown in 2021, the mean wait time to an e-consultation would not have increased, given that the capacity of the unit was increased; hence, the unit would still have been able to meet the demand for e-consultations.

Concerns have been raised that, although e-consultations reduce wait time, the rate of complications may rise. However, this concern is not supported by the results reported in the literature. In a randomized clinical trial conducted in a Unit of Cardiology, Olayiwola et al. observed that the number of admissions to the Emergency Department was lower in the intervention group [23]. In another experience in a Unit of Cardiology, Wasfy et al. confirmed that the e-consultation model is safe and effective and improves the efficacy of outpatient care [27]. Finally, Rey-Aldana et al. implemented an e-consultation model in a Unit of Cardiology in our healthcare district and concluded that this model is safe since it improved waiting time and reduced hospital admissions and mortality during the first year of implementation [6]. In our case, there are no data available in the literature about health outcomes in terms of ED admissions, hospitalizations, and mortality, which we will investigate in future studies.

Considering the results obtained, the use of e-consultations for the management of patients referred from PH to a Unit of Pulmonology is effective in reducing direct and indirect costs incurred by patients and the healthcare system, as compared to the conventional face-to-face visit model. According to a study undertaken to assess the advantages of telemedicine, the cost of an e-consultation is $120 vs. $228 for face-to-face visits. However, these authors did not consider some of the costs that we included in our estimations [28]. Paquette and Lin assessed environmental pollutant emissions resulting from patient travels (carbon dioxide, carbon monoxide, nitric oxide, and volatile organic compounds) [29]. However, unlike in our study, the saving resulting from the reduction of emissions was not calculated in that study.

This study has some limitations. The intervention was implemented in a specific specialty unit, and the impact of this e-consultation model may be different in other specialties [30]. Although our consultations are 100% open since January 1^st^, 2021 without any restrictions at all, limitations in PH, added to patients' reluctance to go to the hospital due to the COVID-19 pandemic, may have contributed to the reduction in the number of consultations. Finally, the occurrence of complications was out of the scope of this study. The data provided about this issue are based on the results provided in the literature.

In summary, the results obtained in this study suggest that an initial evaluation via an e-consultation in outpatient pulmonology care reduces significantly the waiting time and facilitates the identification of less complex cases that do not require a face-to-face visit, whereas it improves access of complex patients to specialty care. This model ensures that clinical problems are managed at the appropriate level of healthcare and contributes to the reduction of healthcare costs. The implementation of this model would improve the efficacy of outpatient pulmonology care.

## Figures and Tables

**Table 1:** Clinical-epidemiological characteristics of patients in the preintervention (2019) and the postintervention (2021) year.

	2019	2021	*p*
Patients (*n*)	7,055	3,934	
Men (%)	52.1	50.2	0.056
Age (years)	60.3 ± 17.2	60.4 ± 17.2	0.820
Previous history (%)			
Heart failure	7.2	6.9	0.541
Atrial fibrillation	9.1	8.7	0.474
Cerebrovascular disease	0.3	0.4	0.344
Pulmonary embolism	1	1.1	0.667
Neoplasm	2.5	2.2	0.352
Chronic obstructive pulmonary disease	21.9	20.4	0.068
Bronchial asthma	18.9	19.1	0.802
Rhinitis	6.8	6.8	0.986
Obesity	27.9	26.5	0.119
Diabetes mellitus	17.6	16.4	0.107

**Table 2:** Annual time (in minutes and hours) spent on e-consultations and first face-to-face visits in the pre and postintervention years and estimated time for the first postintervention year. Average number of weekly consultations (e-consultations, face-to-face visits, and the sum of both) necessary to attend that number of consultations (44 week/year).

*Actual time spent on e-consultations and first face-to-face visits*.								

Year	e-consultations (x15′)	Time minutes (hours)	e-consultations/week (44 week/year)	FtoF visit (x20′)	Time minutes (hours)	FtoF visists/week (44 week/year)	Total minutes (hours)	e-consultations + FtoF/week (44 week/year)

2019	466	6,990′ (116.5 hours)	11	6,589	131,780′ (2,196.3 hours)	150	138,770′ (2,312.8 hours)	161
2021	3,934	59,010′ (983.5 hours)	90	2,223 (56.5%)^*∗*^	44,460′ (741 hours)	51	103,470′ (1,724.5 hours)	141

*Estimates for 2021*								
2021	6,908	103,620′ (1,727 hours)	157	4,145 (60%)^∏^	82,900′ (1,382 hours)	94	186,520′ (3,109 hours)	251

FtoF, face to face; ^*∗*^, percentage with respect to the number of e-consultations for that year; ^∏^, estimated percentage that should be addressed with respect to the number of e-consultations.

**Table 3:** Costs per individual medical procedure and total costs.

Costs	Cost comparison between e-consultations and face-to-face consultations			Total cost of the program	
	e-consultation	Face-to-face visit	e-consultation + face-to-face visit	Type of consultation	Cost (€)
Cost per consultation (€)	105.10	175.10	280.20	Hypothetical cost of the year 2021 if all consultations would have been in person (3,934 consultations × €840.20)	3,305,346.80
Travel costs (€)	0.80^*∗*^	6.20^*∗∗*^	7	Actual total cost of the program: (i) Real cost of the e-consultations completed (1,711 × €187.40) (ii) Real cost of e-consultations + face-to-face visit completed (2,223 × €970.60)	2,478,285.20 320,641.40 2,157,643.80
Time cost (€)^∞^	2.70	8.30	11		
Pollutant emission costs (€)	67.40	564.40	632		
Wait list cost (€)^≠^	11.40	86.20	40.40	Savings resulting from the program	827,061.60
Total cost (€)	187.40	840.20	970.60		
Savings resulting from e-consultations	652.80 €				

^*∗*^ from patient's home to health center. ^*∗∗*^ from patient's home to hospital. ^∞^ depending on the distance to the health center/hospital and their employment situation (active or retired person) (average costs). ^≠^ according to a waiting list of 3.2 days for an e-consultation and 25.7 for a face-to-face consultation (average costs).

## Data Availability

The data used to support the findings of this study were supplied by Luis Valdés under license and so cannot be made freely available. Requests for access to these data should be made to Luis Valdés (Luis.valdes.cuadrado@sergas.es).

## Abbreviations

e-consultation: Electronic consultation
ED: Emergency department
€: Euros
GP: General practitioner
PH: Primary healthcare.
